# Effect of Hot-Pressing Process on Mechanical Properties of UHMWPE Fiber Non-Woven Fabrics

**DOI:** 10.3390/ma17112611

**Published:** 2024-05-28

**Authors:** Jiaxiang Huang, Xiaoping Zhang, Tianyi Gu, Fubao Zhang, Yanfeng Niu, Susu Liu

**Affiliations:** 1 School of Mechanical Engineering, Nantong University, Nantong 226019, Chinazhang.fb@ntu.edu.cn (F.Z.); 2 Jiangsu Xingi High Performance Fiber Products Co., Ltd., Nantong 226400, China

**Keywords:** UHMWPE fiber, non-woven fabric, hot-pressing process, mechanical properties

## Abstract

In order to investigate the influence of a hot-pressing process on the mechanical properties of ultra-high molecular weight polyethylene (UHMWPE) fiber non-woven fabrics with stretch and in-plane shear, UHMWPE non-woven fabric samples were prepared by adjusting the temperature, time, and pressure of the hot-pressing process, and mechanical property tests were carried out so as to clarify the influence of the hot-pressing process on the mechanical properties of the samples. The results show that the hot-pressing process mainly affects the silk–glue bonding strength of the samples; in the test range, with the increase in hot-pressing temperature and time, the tensile strength and in-plane shear strength of the samples increase and then decrease, and the best mechanical properties are obtained at 130 °C and 7 min of hot pressing, respectively; at 130 °C, the in-plane shear strength is 39.94 MPa and the tensile strength is 595.43 MPa; at 7 min, the in-plane shear strength is 63.0 MPa and the tensile strength is 643.30 MPa; with the increase in the hot-pressing pressure, the in-plane shear strength of the samples increases and then decreases, and the highest is 52.60 MPa, achieved at 8 MPa; in the range of 5–8 MPa, the tensile strength of the specimens did not change significantly, and increased significantly at 9 MPa, reaching a maximum strength of 674.55 MPa.

## 1. Introduction

Ultra-high molecular weight polyethylene (UHMWPE) fiber has excellent low-temperature impact durability and chemical resistance, excellent abrasion resistance, minimal moisture absorption, and low density [1,2,3,4,5,6], and the tensile strength is 13 times that of steel and 4 times that of para-aramid fibers, making it one of the strongest fibers in the world [7], which has attracted widespread attention in the field of protection. To avoid the loss of fiber strength during the weaving process, UHMWPE fibers are primarily used in the form of uni-directional (UD) fabric or orthogonally laminated UD fabric, known as non-woven fabric (composed of two layers of UD fabric laminated together), as the constituent elements of composite armor plates [8]. Compared to ordinary fabrics, the fibers in the non-woven fabric are aligned straight without crossing, which allows for rapid energy dispersion during impact, thereby effectively reducing fiber damage. Therefore, UHMWPE fiber non-woven fabric is often used as a component of bulletproof vests or other protective structures. The primary molding process for creating UHMWPE fiber non-woven fabric is hot pressing [9], where the temperature, duration, and pressure of the press significantly influence the mechanical properties of the non-woven fabric [10]. Therefore, extensive research has been conducted globally on the manufacturing processes, mechanical properties, and energy absorption mechanisms of UHMWPE fiber non-woven fabrics.

Cline et al. [11] investigated the relationship between the quasi-static in-plane shear performance and ballistic properties of UHMWPE composite laminates, taking Dyneema HB212 and HB210 as the research objects, both of which are UHMWPE fibers with the same volume fraction and yarn size. However, HB210 has a polyurethane matrix and HB212 has a Kraton^®^ matrix, and they found that Dyneema HB212 exhibits double the in-plane shear strain and a 5% improvement in ballistic limit velocity compared to HB210. Ogawa et al. [12] highlighted the critical impact of hot-pressing temperature on the bonding performance of UHMWPE composites. Karbalaie et al. [13] identified that the optimal anti-impact performance of bulletproof composite laminates is achieved at a curing temperature of 125 °C. Cao et al. [14] evaluated the effect of temperature on the ballistic performance of UHMWPE laminates, noting differences in deformation processes, failure characteristics, and penetration depths across a temperature range from −20 to 95 °C. Zeng et al. [15] reported a decrease in tensile strength and an increase in interface bonding strength with rising hot-pressing temperatures. Zheng et al. [16] assessed the influence of pressure and temperature on the interface bonding strength of UHMWPE fiber/styrene-butadiene-styrene resin composites through T-peel tests, finding a more pronounced effect of pressure over temperature. Comte et al. [17] observed that insufficient hot-pressing pressure could increase void content in composites, thereby degrading the material’s performance. Laessig et al. [18] concluded that higher curing pressures enhance the ballistic performance of UHMWPE bulletproof composites. Forster et al. [19] noted the detrimental effects of extended hot-pressing durations on fiber tensile strength. These findings underscore the significance of processing conditions in optimizing the mechanical properties of UHMWPE fiber composites, particularly emphasizing the sensitivity of these materials to variations in temperature, time, and pressure during hot pressing.

In summary, the hot-pressing process has a significant impact on the performance of UHMWPE fiber composites. Studying the influence patterns and mechanisms of the hot-pressing process on UHMWPE fiber composites is of great significance in the field of protection. However, the influence patterns of the hot-pressing process on the mechanical properties of UHMWPE fiber non-woven fabrics are still not clear enough, and the mechanism research on the hot-pressing process of UHMWPE fiber non-woven fabrics is insufficient. In this paper, tensile and in-plane shear tests are conducted on 2UD non-woven fabrics under various hot-pressing temperatures, times, and pressures. The aim is to investigate the influence of different hot-pressing process parameters on the mechanical properties of non-woven fabrics, reveal the impact mechanism of the hot-pressing process on non-woven fabrics, and provide experimental support and a theoretical basis for the preparation of UHMWPE fiber non-woven fabrics.

## 2. Materials and Methods

### 2.1. Materials

**Figure 1:**
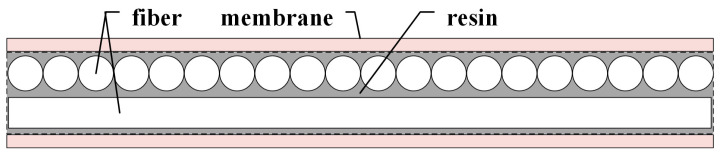
2UD fabric structure diagram.

The non-woven fabric used for the experiments was a UHMWPE fiber thermoplastic resin-based 2UD (Uni-Directional) fabric. The monofilament strength of UHMWPE fiber is 30 cN/dtex, and it is produced by Jiangsu Xingi High Performance Fiber Products Co., Ltd.; the resin matrix is water-based polyurethane, with a solid content of 42.4 and a viscosity of 80.5 MPa·s; the film is a polyester film with a thickness of 3 μm and an areal density of 4 g/m^2^. The 2UD fabric used had an orthogonal structure of 2UD fabric, illustrated in Figure 1. The 2UD fabric had a rubber content of 15%, a surface density of 67 g/m^2^, and a thickness of 0.09 mm. The 2UD fabric was subjected to hot pressing to obtain the test fabric, with the parameters of the hot pressing as shown in Table 1.

### 2.2. Methods

**Figure 2:**
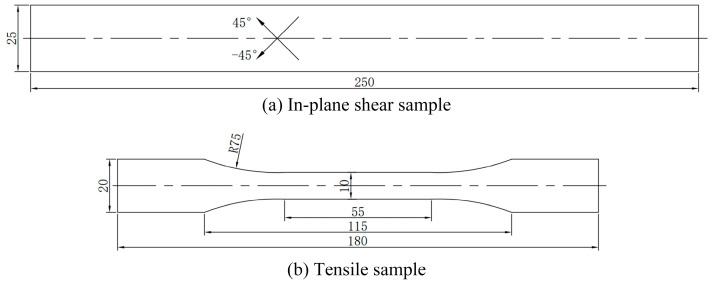
Schematic diagram of the samples (in mm).

The in-plane shear test of the UHMWPE fiber 2UD samples was conducted in accordance with ASTMD3518. The schematic diagram of the in-plane shear sample is shown in Figure 2, and the required samples were obtained using a cold processing method. The testing platform used was the 5969H universal material testing machine, produced by the American company Instron in 2016, with a loading rate of 60 mm/min.

The tensile test of the UHMWPE fiber 2UD samples was conducted in accordance with GB/T1446-2005, “Test Method for Tensile Properties of Fiber Reinforced Plastics” [20]. The schematic diagram of the tensile sample is shown in Figure 2. The sample preparation method and testing method were the same as those used in the in-plane shear test, with a loading speed of 10 mm/min.

Each set of experiments was repeated five times, and the three sets of data with the smallest variance were selected for analysis.

## 3. Results

### 3.1. The Impact of Hot-Pressing Parameters on the In-Plane Shear Properties of 2UD Fabrics

**Figure 3:**
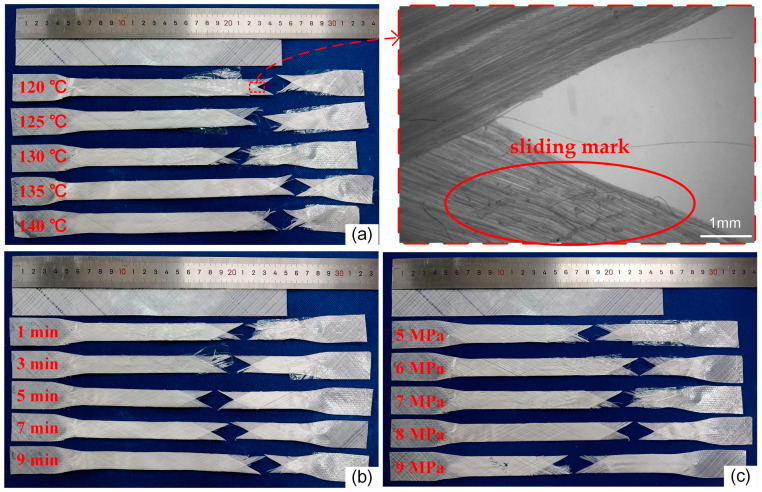
Failure appearance of in-plane shear of samples under different hot-pressing parameters: (**a**) hot-pressing time is 1 min, hot-pressing pressure is 7 MPa; (**b**) hot-pressing temperature is 130 °C, hot-pressing pressure is 7 MPa; (**c**) hot-pressing temperature is 130 °C, hot-pressing time is 1 min.

The damage morphology of the samples after the in-plane shear test under different hot-pressing parameters is shown in Figure 3. From the figure, the samples exhibit significant shrinkage and elongation in both width and length directions. At the fracture sites of the samples, evident relative slipping marks between the fibers and the resin matrix can be observed, along with a small amount of fiber pull-out. The primary load-bearing entities during the in-plane shear tests are the resin matrix and the interfacial bonding strength between the fiber and resin. Among the three groups of samples, membrane–fiber separation phenomena occurred when the hot-pressing temperature was below 135 °C, the hot-pressing time was less than 5 min, and the hot-pressing pressure was below 8 MPa. During the in-plane shear of the sample, the deformation ability of the film is inconsistent with that of the resin, resulting in the separation of the film from the non-woven fabrics when the sample is deformed. However, this phenomenon gradually disappears with the increase in the hot-pressing parameter. This is because the viscosity of the resin increases with the increase in the hot-pressing parameter, making the film stick to the non-woven fabrics better. This indicates that the increase in hot-pressing temperature, hot-pressing time, and hot-pressing pressure can improve the bonding property of resin to a certain extent, and thus increase the film–fiber bonding strength.

**Figure 4:**
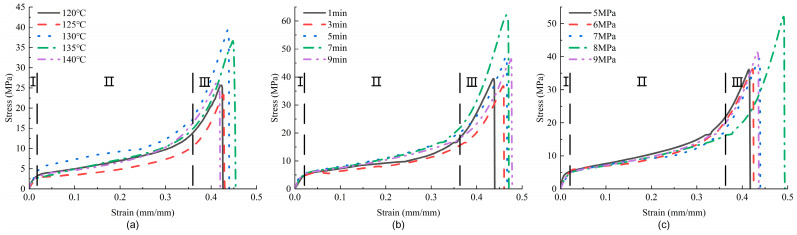
In-plane shear stress–strain curves of samples under different hot-pressing parameters: (**a**) hot-pressing time is 1 min, hot-pressing pressure is 7 MPa; (**b**) hot-pressing temperature is 130 °C, hot-pressing pressure is 7 MPa; (**c**) hot-pressing temperature is 130 °C, hot-pressing time is 1 min.

**Figure 5:**
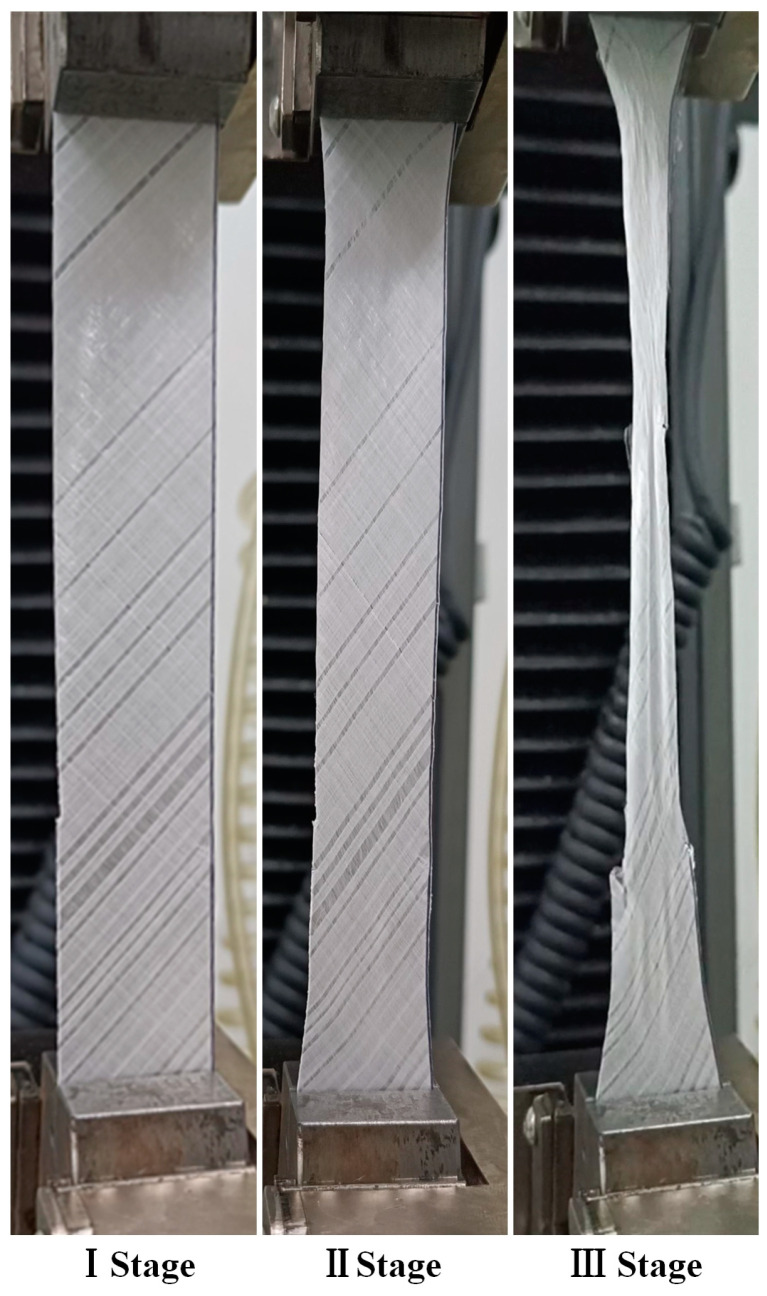
Sample morphologies at three stages during in-plane shear testing.

The stress–strain curves of the in-plane shear of the samples with different hot-pressing parameters are shown in Figure 4. The representative curve for each sample is selected based on the ultimate stress and ultimate strain, which are close to the average experimental data. As can be observed from the figure, all curves exhibit a similar change process, which can be divided into three stages: The first stage is elastic deformation, where the curve shows a linear relationship and occupies the shortest proportion during the test. The second stage is the yield stage, which takes up the longest proportion during the test, the load increases slowly, and the curve is relatively flat, indicating that the sample primarily withstands the load through its own deformation (gradually narrowing width and decreasing fiber angle). The third stage is the plastic strengthening stage, where the stress on the sample rapidly rises in a short time until instantaneous fracture. The morphology of the samples at the three stages is shown in Figure 5.

Observing the curves of the three groups, Figure 4a shows the greatest degree of dispersion, being more consistent only in the first stage. This suggests that changes in hot-pressing temperature not only alter the bond strength between the thread and the glue in the samples but may also cause varying degrees of damage to the fibers in the samples, leading to significant differences in the stress–strain curves under different hot-pressing temperatures. The in-plane shear performance of this group of samples is better at 130 °C and 135 °C. The fluctuations of the curve in Figure 4b have reduced, with the first stage almost completely overlapping. At 7 min, the peak of the curve significantly increases, obtaining better in-plane shear performance. The curves in Figure 4c showed a high degree of consistency, with strain and peak values significantly increasing at 8 MPa, indicating the best in-plane shear performance during the test. The ultimate strain of the curves reflects the samples’ elastic properties, which change with the hot-pressing parameters. As the hot-pressing parameters increase, the strain of the three groups of curves first increases and then decreases. The size of the enclosed area formed by the curves and the *x*-axis reflects the amount of energy absorbed by the samples, revealing a trend similar to the stress.

**Figure 6:**
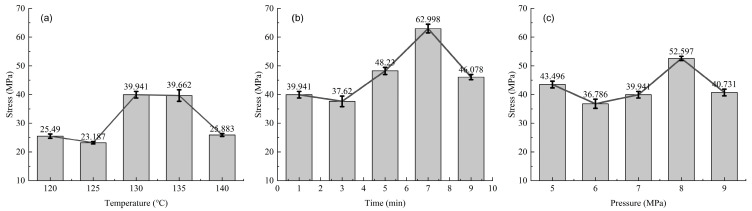
In-plane shear strength of samples under different hot-pressing parameters: (**a**) hot-pressing time is 1 min, hot-pressing pressure is 7 MPa; (**b**) hot-pressing temperature is 130 °C, hot-pressing pressure is 7 MPa; (**c**) hot-pressing temperature is 130 °C, hot-pressing time is 1 min.

The strength of the samples under different hot-pressing parameters during the in-plane shear test is shown in Figure 6. The changing pattern of the three groups of lines is the same, decreasing first, then increasing, and finally decreasing again. Figure 6a has a generally lower strength, with the highest in-plane shear strength at 130 °C being 39.94 MPa, and the lowest, at 125 °C, being 23.19 MPa, with a range of 16.75 MPa, showing a 72.3% increase from the lowest value to the highest. Figure 6b samples have the highest in-plane shear strength at 7 min, which is also the highest value among the three groups of tests, being 63.0 MPa; the lowest is at 3 min, being 37.62 MPa; and the range is 25.38 MPa, showing a 67.5% increase from the lowest value to the highest. Figure 6c has the highest in-plane shear strength at 8 MPa, of 52.60 MPa; the lowest is at 6 MPa, of 36.79 MPa; and the range is 15.81 MPa, showing a 43.0% increase from the lowest value to the highest.

A one-way analysis of variance was conducted on the in-plane shear test results to observe the significance of the influence of each thermal parameter on the in-plane shear strength of the sample. The analysis results are shown in Table 2. As can be seen from the table, when the confidence coefficient α is set to 0.05, the analysis results of the three groups of tests all show a *p*-value of ≤0.01 and F ≥ F-crit, indicating that the three hot-pressing parameters have extremely significant effects on the in-plane shear properties of the samples.

**Figure 7:**
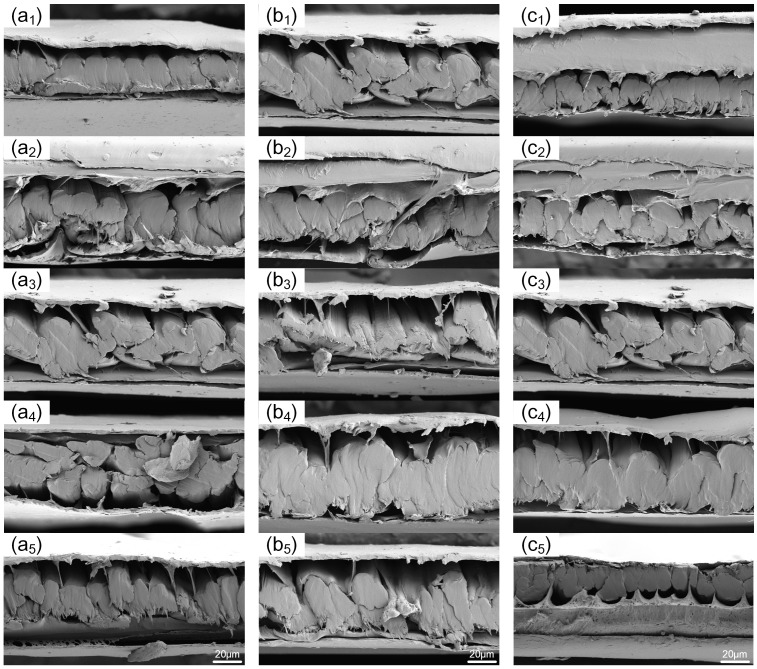
Cross-section of samples under different hot-pressing parameters: (**a_1_**–**a_5_**) hot-pressing time is 1 min, hot-pressing pressure is 7 MPa, and hot-pressing temperature is 120 °C, 125 °C, 130 °C, 135 °C, and 140 °C in sequence; (**b_1_**–**b_5_**) hot-pressing temperature is 130 °C, hot-pressing pressure is 7 MPa, and hot-pressing time is 1 min, 3 min, 5 min, 7 min, and 9 min in sequence; (**c_1_**–**c_5_**) hot-pressing temperature is 130 °C, hot-pressing time is 1 min, and hot-pressing pressure is 5 MPa, 6 MPa, 7 MPa, 8 MPa, and 9 MPa in sequence.

Since the main failure mode of in-plane shear is the relative slippage between the resin and fiber layers, scanning electron microscopy was used to observe the microscopic morphology of sample cross-sections under different hot-pressing processes. This was performed to investigate the variation in the degree of sericin bonding in samples under different hot-pressing parameters and to thereby explore the mechanism behind the changes in mechanical properties of the samples when the hot-pressing parameters are altered. The observation results are presented in Figure 7. Due to the cross-sections being formed by shearing, some transverse fibers were squeezed and separated under the action of shearing forces, leading to some samples having no transverse fibers visible and separating from the film. However, this did not affect the bonding between the sheared fibers and the resin. Analysis revealed that the fibers, encased in resin, formed bundles of varying sizes, with gaps of different sizes existing between these bundles. As the three parameters increased, the cross-sectional area of the fiber bundles generally showed a trend of first increasing and then decreasing. This further indicates that changes in hot-pressing parameters significantly alter the degree of bonding between UHMWPE fiber bundles, thus affecting the fiber–resin bond strength.

When changing the hot-pressing temperature, the sample at 125 °C compared to its adjacent samples showed less orderly arranged fiber bundles and more gaps between them; at 130 °C, the fiber–resin integrity was the highest, with larger cross-sections of fiber bundles formed by bonding between fibers. With the increase in the three hot-pressing parameters, the cross-sectional area of the fiber bundle formed by resin encapsulation generally exhibits a trend of first increasing and then decreasing. When altering the hot-pressing time, the sample at 3 min showed a lower degree of bonding of fiber bundles compared to its group’s adjacent samples, while, at 7 min, the fiber bundles achieved the best adhesive effect, forming large integrated areas with a cross-sectional area significantly higher than other groups. Changing the hot-pressing pressure, the sample at 6 MPa showed larger gaps between each fiber bundle, with a bonding effect significantly differing from other samples in the group. The sample at 8 MPa exhibited a better bonding effect of fiber bundles, with the largest integrated areas and the least gaps, and the fiber bundle bonding conditions of the other three samples in the pressure group were similar. The results of the in-plane shear test were consistent with the trends in the change in the fiber–resin bonding situation in the sample cross-sections, with the in-plane shear strength reflecting, to a certain extent, the degree of fiber–resin bonding.

### 3.2. The Impact of Hot-Pressing Parameters on the Tensile Properties of 2UD Fabric

**Figure 8:**
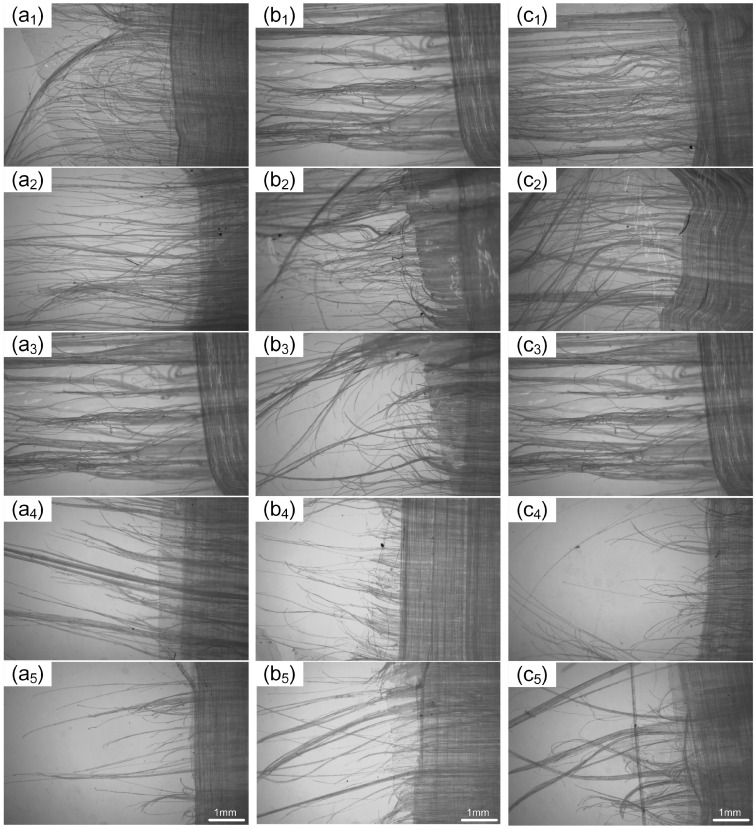
Tensile failure appearance of samples under different hot-pressing parameters: (**a_1_**–**a_5_**) hot-pressing time is 1 min, hot-pressing pressure is 7 MPa, and hot-pressing temperature is 120 °C, 125 °C, 130 °C, 135 °C, and 140 °C in sequence; (**b_1_**–**b_5_**) hot-pressing temperature is 130 °C, hot-pressing pressure is 7 MPa, and hot-pressing time is 1 min, 3 min, 5 min, 7 min, and 9 min in sequence; (**c_1_**–**c_5_**) hot-pressing temperature is 130 °C, hot-pressing time is 1 min, and hot-pressing pressure is 5 MPa, 6 MPa, 7 MPa, 8 MPa, and 9 MPa in sequence.

The failure morphology at the fracture sites of tensile samples under different hot-pressing parameters is shown in Figure 8. Due to the orthogonal 2UD structure of the samples, damage at the fracture sites manifests as longitudinal fiber fractures and pull-outs in the direction of the load, and lateral separation and plastic deformation between the resin and fibers. The fractures typically occur at the weakest part of the sample, influenced by the uniformity of fiber distribution and hot-pressing parameters, resulting in varied weaknesses across individual fibers and, hence, differing degrees of fiber pull-out at the fracture sites. As the three hot-pressing parameters increase, the number of pulled-out fibers at the fracture sites shows varying degrees of reduction; simultaneously, the amount of resin attached to the pulled-out fibers also differs discernible when observing the adhesion of the pulled-out fibers.

The fractures in all three sample groups tended to become neater with the increase in the parameters, especially at 140 °C, 1 min, 7 MPa; 130 °C, 7 min, 7 MPa; and 130 °C, 1 min, 8 MPa; these conditions resulted in the neatest fractures. In the early stages of parameter variation, the amount of fiber pull-out changed little, but the amount of resin adhering to the pulled-out fibers showed more noticeable changes. In the three experimental groups, the least amount of resin on the pulled-out fibers occurred, respectively, in Figure 8(a_2_,b_2_,c_2_), where the fibers appeared thread-like with less adhesion. Against the samples in Figure 8, it is evident that, with the increase in fiber–resin bond strength, both the number of pulled-out fibers and the amount of attached resin decrease, enhancing the neatness of the tensile fracture; however, in Figure 8(a_4_,a_5_), the neatness of the fractures is inversely related to the changes in fiber–resin bond strength. Particularly, in Figure 8(a_5_), despite the lower fiber–resin bond strength, the fracture appears neatest. Considering that the melting point of the fibers is between 130 and 136 °C, and the hot-pressing temperature in Figure 8(a_4_,a_5_) falls within this melting range, the excessively high temperature can cause fiber damage or partial melting, which reduces the tensile strength of the fibers making it difficult for them to pull out from the resin, thus affecting the degree of fiber pull-out at the fracture site.

**Figure 9:**
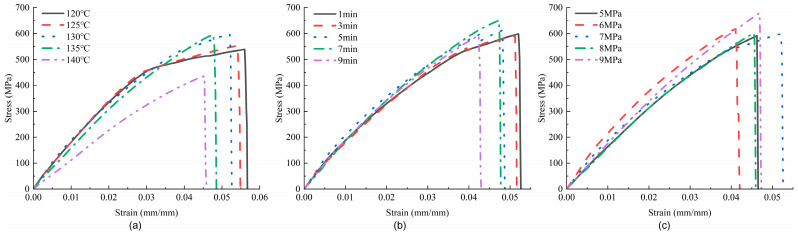
Tensile stress–strain curves of samples under different hot-pressing parameters: (**a**) hot-pressing time is 1 min, hot-pressing pressure is 7 MPa; (**b**) hot-pressing temperature is 130 °C, hot-pressing pressure is 7 MPa; (**c**) hot-pressing temperature is 130 °C, hot-pressing time is 1 min.

The stress–strain curves under different hot-pressing parameters for tensile testing are displayed in Figure 9. The trend of the curves is consistent, with Figure 9b showing the best consistency and Figure 9a displaying the greatest dispersion. For both Figure 9a,b, the strain decreases with increasing parameters, while the peak first increases and then decreases. The enclosed area formed by the curves and the *x*-axis reflects the energy absorption of the samples. When both the strain and peak values decline, the overall performance of the samples also decreases. For Figure 9c, the smallest tensile strain occurs at 6 MPa and the largest at 7 MPa, with smaller differences among the other samples; the energy absorption trend for all three groups aligns with the trend of maximum strain changes. Compared to the in-plane shear curves, the strain values in the tensile curves are significantly reduced, and the peaks are noticeably increased. While in-plane shear performance mainly relates to the strength of the resin and fiber–resin bond strength, tensile performance primarily reflects the degree of fiber deformation (elastic properties) and tensile characteristics. Therefore, a reduction in energy absorption can indicate a degradation in fiber performance to some extent. When the hot-pressing temperature reaches 140 °C, the sample’s ability to absorb energy significantly declines, notably lower than at other temperatures. This supports the analysis that at this temperature, the fibers are damaged due to excessive heat, corroborating the notion that tensile test data can reflect changes in fiber performance to some degree.

**Figure 10:**
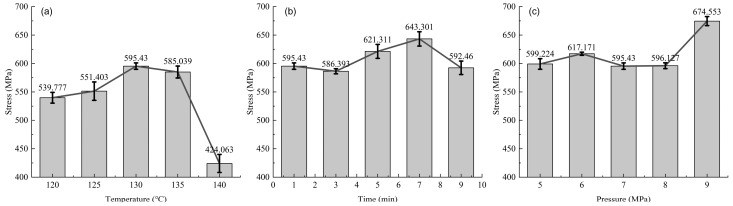
Tensile strength of samples under different hot-pressing parameters: (**a**) hot-pressing time is 1 min, hot-pressing pressure is 7 MPa; (**b**) hot-pressing temperature is 130 °C, hot-pressing pressure is 7 MPa; (**c**) hot-pressing temperature is 130 °C, hot-pressing time is 1 min.

The tensile strength under different hot-pressing parameters is shown in Figure 10. The tensile strength of the samples increases and then decreases with the increase in hot-pressing temperature and time. In Figure 10a, the maximum strength is at 130 °C, reaching 595.43 MPa, while the minimum is at 140 °C, at 424.06 MPa; this represents a range of 171.37 MPa, with the maximum value being 40.4% higher than the lowest. In Figure 10b, the maximum strength occurs at 7 min, at 643.30 MPa, and the minimum at 3 min, at 586.39 MPa; the range is 56.39 MPa, with the maximum value being 9.7% higher than the lowest. For Figure 10c, the tensile strength changes little within the 5–8 MPa range, with a significant increase evident only at 9 MPa. At this pressure, the maximum strength is 674.55 MPa, with the minimum at 7 MPa being 595.43 MPa; the range here is 79.12 MPa, with the maximum value being 13.3% higher than the lowest.

Comparing these trends with the in-plane shear test results, the changes in Figure 10a,b show a similar trend to those of in-plane shear, especially with Figure 10b, where the range is small, suggesting that, within the tested range, hot-pressing time does not significantly affect fiber performance. The changes in strength in Figure 10b could be attributed to changes in fiber–resin bond strength. Figure 10a also shows insignificant changes up to 135 °C, and a substantial decrease occurs at 140 °C due to the degradation of fiber performance, leading to reduced tensile performance. The trend in Figure 10c is different from that of the in-plane shear, showing a significant disparity at 9 MPa. Here, the influence of fiber–resin bond strength on tensile performance is minimal, with fiber fracture playing a dominant role. Between 5–8 MPa, the hot-pressing pressure has little effect on the fibers, but at 9 MPa, it enhances fiber performance. This analysis indicates that the hot-pressing parameters not only affect the physical bonding but also directly influence the intrinsic mechanical properties of the fibers, particularly at higher pressures and temperatures where the risk of fiber damage increases.

A one-way analysis of variance was performed on the tensile test results to observe the significance of the influence of each thermal parameter on the tensile strength of the sample. The analysis results are shown in Table 3. As can be seen from the table, when the confidence coefficient α is set to 0.05, the analysis results of the three groups of tests all show that the *p*-value is ≤0.01, and F ≥ F-crit, indicating that the three hot-pressing parameters have extremely significant effects on the tensile properties of the samples.

## 4. Discussion

### 4.1. The Impact of Hot-Pressing Parameters on In-Plane Shear Properties

As seen from Figure 4, Figure 6 and Figure 7 and Table 2, changes in hot-pressing temperature, time, and pressure all have significant effects on the in-plane shear properties of non-woven fabrics. As the hot-pressing parameters increase, the in-plane shear strength of the samples first decreases, then increases, and finally decreases again, reaching its maximum at 130 °C, 7 min, and 8 MPa, respectively. According to the damage morphology of the samples, the main failure mode of in-plane shear is the relative slippage between resin and fibers. Further, SEM images show that the overall degree of fiber–resin bonding in the three groups of samples first increases and then decreases, which is consistent with the trend of changes in the in-plane shear strength of the samples. This indicates that the in-plane shear properties of the samples are mainly related to the fiber–resin bonding strength. As the hot-pressing temperature, time, and pressure increase, the fluidity of the resin also increases, enabling the resin to fill the gaps between fibers better, reducing porosity, improving the degree of fiber–resin bonding, and thus enhancing the mechanical properties. However, when the hot-pressing temperature is too high or the hot-pressing time is too long, it can lead to degradation and aging of the resin matrix, reducing the mechanical properties [10,21].

The analysis of experimental data indicates that the degree of influence of hot-pressing parameters on the in-plane shear properties of UHMWPE fiber non-woven fabric is temperature > time > pressure.

### 4.2. The Impact of Hot-Pressing Parameters on Tensile Properties

As seen from Figure 8, Figure 9 and Figure 10, and Table 3, changes in hot-pressing temperature, time, and pressure all have significant effects on the tensile properties of non-woven fabrics. With the increase in hot-pressing temperature and time, the tensile strength of the samples first increases and then decreases; while, with the increase in hot-pressing pressure, the tensile strength first increases, then decreases, and finally increases again, reaching its maximum at 130 °C, 7 min, and 9 MPa, respectively. Observing the tensile damage morphology of the samples, it can be seen that the main tensile failure modes of non-woven fabrics are fiber fracture and pull-out. Comparing Figure 8 with Figure 7, it is found that the fracture morphology of the samples is influenced by the degree of sericin bonding, indicating that the tensile properties of non-woven fabrics are mainly related to fiber strength and fiber–resin bonding strength.

Comparing the trends of tensile strength and in-plane shear properties, we can observe that, when the hot-pressing temperature and time vary, the two trends are relatively similar, especially when the hot-pressing time differs with a small range of variation. It can be concluded that, within the experimental range, the hot-pressing time has an insignificant impact on the fiber properties, and the changes in tensile strength may be caused by the variation in fiber–resin bonding strength. With the increase in hot-pressing temperature and time, the crystallinity of fibers will increase accordingly [22], enhancing the fiber strength. However, excessive crystallinity will make the specimen brittle, affecting its mechanical properties. When the hot-pressing temperature exceeds 135 °C, the tensile strength of the specimen decreases significantly because the temperature exceeds the melting point of fibers, damaging the fibers and reducing the tensile performance of the specimen [23]. When the hot-pressing pressure changes, the trend of tensile strength differs from that of in-plane shear, indicating that the influence of fiber–resin bonding strength on tensile strength is relatively small. Between 5 and 8 MPa, the change in tensile strength of the samples is relatively small, and it only increases significantly at 9 MPa. This may be attributed to the fact that when the hot-pressing pressure increases to a certain value it improves the thermal stability of the fibers, resulting in better mechanical properties [24,25].

The analysis of experimental data indicates that the degree of influence of hot-pressing parameters on the tensile properties of non-woven fabrics is temperature > pressure > time.

## 5. Conclusions

This study primarily investigated the effects of hot-pressing temperature, time, and pressure on the in-plane shear and tensile strength of UHMWPE fiber non-woven fabric, yielding the following conclusions:

(a) Within a certain range, as the hot-pressing parameters increase, the fiber–resin bond strength of the non-woven fabric also increases. The in-plane shear performance of the samples is mainly related to this bond strength, while the tensile performance is influenced by both the bond strength and the fibers’ intrinsic properties. Changes in hot-pressing parameters create significant differences in the neatness of the tensile sample fractures and the extent of fiber pull-out from the matrix.
(b) When the hot-pressing time is set to 1 min and pressure to 7 MPa, with temperatures varying between 120 °C and 140 °C, both the tensile and in-plane shear strengths of the non-woven fabric initially increase and then decrease, peaking at 130 °C. The in-plane shear strength changes by 72.3%, and the tensile strength changes by 40.4%.
(c) With a hot-pressing temperature of 130 °C, a pressure of 7 MPa, and time varying from 1 to 9 min, both the tensile and in-plane shear strengths of the non-woven fabric first decrease, then increase and decrease again, reaching their maximum at 7 min. The in-plane shear strength changes by 67.5%, and the tensile strength changes by 9.7%.
(d) At a hot-pressing temperature of 130 °C, time of 1 min, and pressure varying from 5 to 9 MPa, the in-plane shear strength of the non-woven fabric initially decreases, then increases, and decreases again, peaking at 8 MPa. The tensile strength shows no significant change within the range from 5 to 8 MPa but reaches its maximum at 9 MPa. The in-plane shear strength changes by 43%, and the tensile strength changes by 13.3%.

## Figures and Tables

**Table 1:** Hot-pressing process.

Hot-Pressing Parameters	Group Number				
	1	2	3	4	5
Temperature/°C	120	125	130	135	140
Time/min	1	1	1	1	1
Pressure/MPa	7	7	7	7	7
Temperature/°C	130	130	130	130	130
Time/min	1	3	5	7	9
Pressure/MPa	7	7	7	7	7
Temperature/°C	130	130	130	130	130
Time/min	1	1	1	1	1
Pressure/MPa	5	6	7	8	9

**Table 2:** Results of one-way ANOVA of in-plane shear test.

Group	Number	Average/MPa	Variance	F	*p*-Value	F-Crit
120 °C	3	25.490	0.764	108.068	3.479 × 10^−8^	3.478
125 °C	3	23.187	0.231			
130 °C	3	39.940	1.961			
135 °C	3	39.662	6.140			
140 °C	3	25.883	0.358			
1 min	3	39.940	1.961	104.738	4.051 × 10^−8^	3.478
3 min	3	37.620	5.247			
5 min	3	48.230	2.325			
7 min	3	62.998	3.420			
9 min	3	46.078	1.229			
5 MPa	3	43.496	2.077	50.731	1.317 × 10^−6^	3.478
6 MPa	3	36.786	3.789			
7 MPa	3	39.940	1.961			
8 MPa	3	52.597	0.825			
9 MPa	3	40.731	2.070			

**Table 3:** One-way ANOVA of tensile test.

Group	Number	Average/MPa	Variance	F	*p*-Value	F-Crit
120 °C	3	539.777	178.939	47.028	1.883 × 10^−6^	3.478
125 °C	3	551.403	520.797			
130 °C	3	595.430	64.203			
135 °C	3	585.039	221.563			
140 °C	3	424.063	503.129			
1 min	3	595.430	64.203	8.562	0.00287	3.478
3 min	3	586.393	39.714			
5 min	3	621.312	302.242			
7 min	3	643.301	317.986			
9 min	3	592.459	278.659			
5 MPa	3	599.224	174.976	39.251	4.388 × 10^−6^	3.478
6 MPa	3	617.171	14.732			
7 MPa	3	595.430	64.203			
8 MPa	3	596.127	52.817			
9 MPa	3	674.554	125.879			

## Data Availability

Data are contained within the article.
